# Unmasking an Incidental *Trichuris trichiura* Infection in a Patient With Acute Hepatobiliary and Gastrointestinal Illness

**DOI:** 10.1002/jgh3.70146

**Published:** 2025-04-02

**Authors:** Yung‐Che Chou, Yu‐Ta Lin, Tze‐Kiong Er

**Affiliations:** ^1^ Division of Laboratory Medicine, Asia University Hospital Asia University Taichung Taiwan; ^2^ Division of Gastroenterology Asia University Hospital Taichung Taiwan; ^3^ Department of Medical Laboratory Science and Biotechnology Asia University Taichung Taiwan

**Keywords:** colonoscopy findings, gastrointestinal parasitic infection, hepatobiliary dysfunction, incidental parasite detection, *Trichuris trichiura*

## Abstract

**Background:**

*Trichuris trichiura* is a common intestinal parasite, but its systemic impact remains unclear. While severe infections may cause gastrointestinal complications, hepatobiliary involvement is rare. This case describes an incidental *T. trichiura* infection identified during colonoscopy in a patient hospitalized with acute pancreatitis and suspected gastric outlet obstruction. The study underscores the importance of differentiating incidental parasitic infections from true pathology to prevent misdiagnosis and unnecessary treatment.

**Case Presentation:**

A 52‐year‐old female presented with persistent nausea, vomiting, postprandial discomfort, and weight loss for 2 weeks. Imaging revealed hepatomegaly and gastric distension, raising concerns for gastric outlet obstruction or severe gastritis. Laboratory findings showed elevated liver enzymes (ALT: 101 IU/L, Alk‐P: 189 IU/L, r‐GT: 288 U/L). A viral etiology was suspected but not confirmed. The patient received supportive intravenous therapy, and her symptoms resolved. Colonoscopy revealed a partially clamped *T. trichiura* adult worm in the transverse colon. Given her clinical improvement without anthelmintic treatment, the infection was deemed incidental and not causative.

**Conclusion:**

This case highlights the need to critically evaluate incidental parasitic infections before attributing them to clinical symptoms. Routine screening is valuable but should be accompanied by a thorough assessment of parasite burden, patient history, and clinical presentation to guide appropriate management and prevent unnecessary interventions.

## 1 Background

*Trichuris trichiura*, commonly known as whipworm, is a widespread parasite in warm, humid regions, particularly in tropical and subtropical areas [1]. Recently, a systematic review and meta‐analysis estimated that approximately 513 million people worldwide are infected with *T. trichiura* [2]. Infection occurs through the ingestion of embryonated eggs found in contaminated soil or food, potentially leading to chronic infection [3]. The severity of symptoms correlates directly with the worm burden in the gastrointestinal tract [4]. Although rare, heavy infections can lead to severe complications such as chronic diarrhea, iron deficiency anemia, and, in extreme cases, Trichuris dysentery syndrome. Although *T. trichiura* remains endemic in many tropical and subtropical regions, its prevalence has significantly declined in areas with improved sanitation and healthcare access. However, sporadic cases still occur, particularly among individuals with a travel history to endemic areas or compromised immune function, making incidental infections relevant even in regions like Taiwan, where public health measures have reduced overall prevalence [5].

## 2 Case Presentation

We present the case of a 52‐year‐old female admitted in November 2024 with acute pancreatitis, abnormal liver function tests, delayed gastric emptying, and splenomegaly. She reported persistent nausea, vomiting, postprandial discomfort, and significant weight loss over the past 2 weeks. Initial imaging revealed hepatomegaly and gastric distension, raising concerns for gastric outlet obstruction or severe gastritis. Laboratory findings showed elevated ALT (101 IU/L), alkaline phosphatase (189 IU/L), and gamma‐glutamyl transferase (r‐GT, 288 U/L), which suggests hepatobiliary involvement.

Given the constellation of symptoms, an acute viral infection was suspected as the underlying cause, though no specific pathogen was identified. The patient received supportive intravenous fluid therapy without targeted pharmacological intervention, and her symptoms gradually resolved. Since *T. trichiura* is not commonly associated with hepatobiliary abnormalities and the patient improved without anthelmintic treatment, the parasitic infection was considered incidental rather than the primary etiology. Many *T. trichiura* infections remain asymptomatic, as clinical disease is largely dependent on parasite burden [3]. It is possible that our patient had a low parasite load, contributing to the absence of significant gastrointestinal symptoms.

**FIGURE 1:**
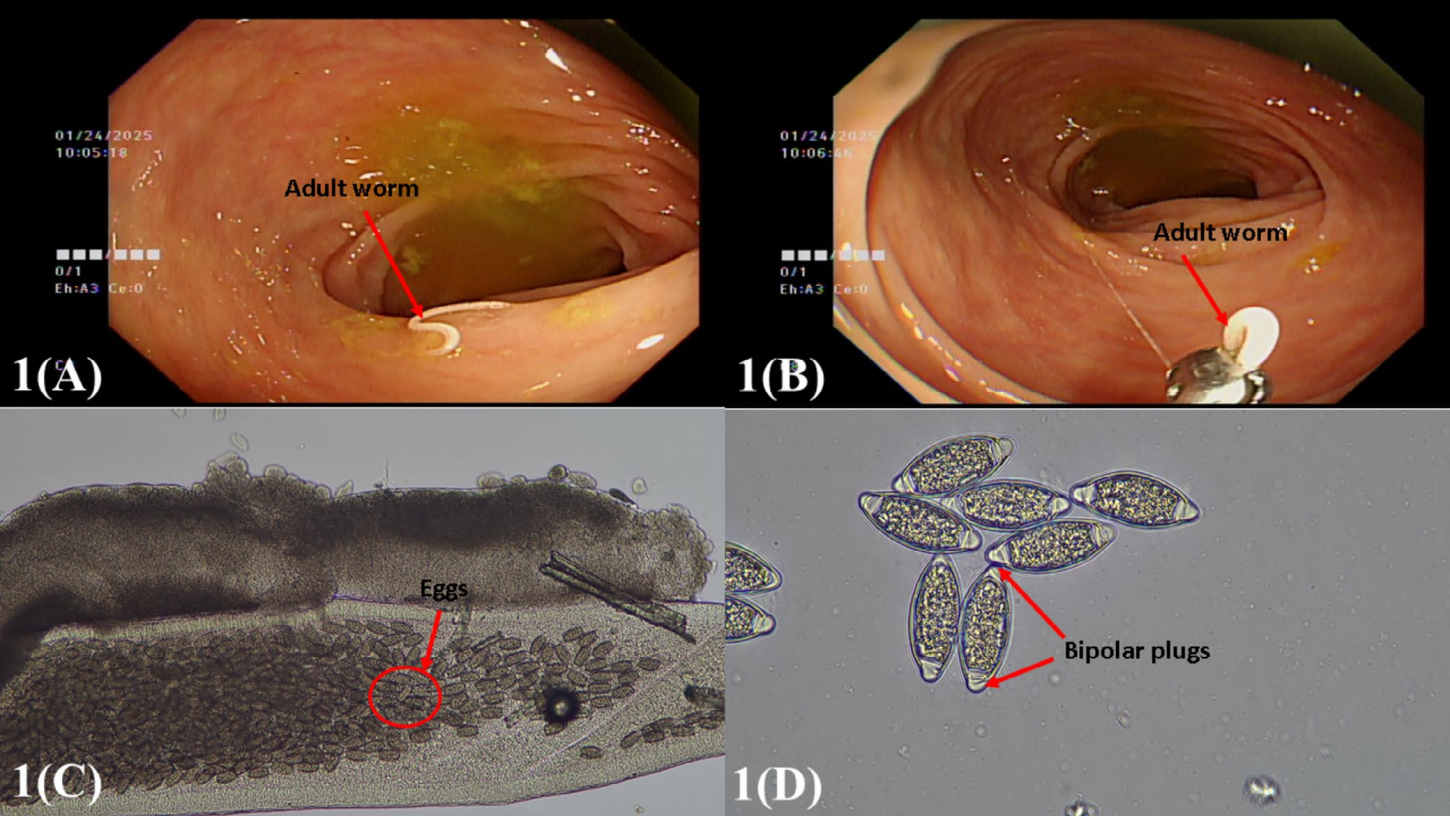
Representative images of *Trichuris trichiura* infection. (A) Colonoscopic image of an adult *T. trichiura* worm in the transverse colon. (B) Closer view of *T. trichiura* showing partial clamping of its body. (C) Microscopic image of a fragmented *T. trichiura* worm with numerous visible eggs (magnification: 40×). (D) Direct microscopic examination confirms *T. trichiura* infection, highlighting its characteristic barrel‐shaped eggs with bipolar plugs (magnification: 100×).

During a subsequent colonoscopy, an adult *T. trichiura* worm was identified in the transverse colon, with its body partially clamped (Figure 1A,B). Numerous visible eggs were observed within the fragmented worm (Figure 1C). Biopsy samples were examined using light microscopy with a saline wet mount preparation, confirming *T. trichiura* infection (Figure 1D). However, given the patient's clinical improvement without anthelmintic treatment and the lack of evidence linking *T. trichiura* to hepatobiliary abnormalities, the parasitic infection was deemed an incidental finding rather than the primary etiology of her symptoms.

## 3 Discussion

Although *T. trichiura* primarily resides in the large intestine, its potential systemic effects remain uncertain. Reports of hepatobiliary involvement are rare, and direct hepatic invasion is not a recognized feature of its lifecycle. However, chronic parasitic infections can modulate host immune responses, potentially leading to systemic inflammation that may indirectly influence hepatobiliary function [6]. Though this remains theoretical in humans, *Trichuris muris* infection models suggest that prolonged immune activation may have extragastrointestinal effects.

Comparative case studies, such as the one by Azira and Zeehaida [4], demonstrate that high parasite burdens are strongly associated with severe gastrointestinal distress and chronic iron deficiency anemia, particularly in pediatric patients with Trichuris dysentery syndrome. In contrast, low‐burden infections—as seen in this case—are often asymptomatic and should be carefully assessed before being considered clinically significant.

Behniafar et al. conducted a meta‐analysis on the global prevalence of *T. trichiura*, revealing a pooled prevalence between 6.64% and 7.57%, with the highest rates observed in the Caribbean and South‐East Asia [2]. Their findings highlight the persistent global health challenge of *T. trichiura* infections, emphasizing the need for targeted control measures rather than indiscriminate treatment.

Additionally, co‐infections involving *T. trichiura* have been reported to exacerbate hepatopathology in experimental models. A study on baboons demonstrated that chronic *T. trichiura* infection intensified liver pathology caused by *Schistosoma mansoni* eggs, suggesting that whipworm infections may worsen hepatic conditions in co‐infected hosts [7]. While such findings remain largely experimental, they underscore the potential for helminthic interactions to contribute to systemic inflammation, necessitating further investigation into their clinical implications.

Furthermore, Hsu and Fan [5] conducted a retrospective study on emerging and reemerging parasitic diseases in Taiwan, revealing that nematode infections accounted for 46.97% of cases, particularly in areas with potential exposure to contaminated soil or food. Their findings highlight the importance of considering parasitic infections in patients with unexplained gastrointestinal symptoms, especially in endemic or high‐risk populations.

This case underscores the diagnostic complexity of overlapping gastrointestinal and hepatobiliary symptoms and highlights the importance of distinguishing incidental findings from primary pathology. Further research is warranted to determine whether chronic Trichuris infections contribute to systemic inflammatory processes that could affect liver function in specific populations.

## 4 Conclusion

This case reinforces the need to critically assess incidental parasitic infections in patients with unexplained gastrointestinal symptoms. Routine screening alone does not justify treatment unless a high parasite burden, persistent symptoms, or immunocompromised status necessitate intervention. Clinicians should consider patient history, parasite load, and coexisting conditions before determining the clinical significance of incidental findings. A thorough correlation of parasitic findings with imaging and laboratory data is essential to prevent misattribution of symptoms and unnecessary treatment. Further evaluation should be considered when patients present with persistent gastrointestinal distress, evidence of malabsorption, or ongoing hepatobiliary dysfunction.

## Ethics Statement

The authors have nothing to report.

## Consent

The authors have nothing to report.

## Conflicts of Interest

The authors declare no conflicts of interest.

## Data Availability

The data supporting the findings of this study are included within the manuscript.
